# Impact of diastolic pulmonary gradient and pulmonary vascular remodeling on survival after left ventricular assist device implantation and heart transplantation

**DOI:** 10.1186/s43044-023-00428-4

**Published:** 2023-12-20

**Authors:** Mohamed Laimoud, Emad Hakami, Mary Jane Maghirang, Tahir Mohamed

**Affiliations:** 1 Cardiovascular Critical Care Department, King Faisal Specialist Hospital and Research Center, Riyadh, Saudi Arabia; 2 Critical Care Medicine Department, Cairo University, Cairo, Egypt; 3 Cardiovascular Nursing Department, King Faisal Specialist Hospital and Research Center, Riyadh, Saudi Arabia; 4 Cardiovascular Medicine Department, King Faisal Specialist Hospital and Research Center, Riyadh, Saudi Arabia

**Keywords:** Diastolic pulmonary gradient (DPG), Pulmonary vascular resistance (PVR), Transpulmonary gradient (TPG), Heart transplantation, Left ventricular assist device (LVAD), HeartMate III, Right ventricular failure (RVF), Mortality

## Abstract

**Background:**

The left ventricular assist devices (LVADs) are increasingly used for advanced heart failure as a bridge to heart transplantation or as a destination therapy. The aim of this study was to investigate the changes of diastolic pulmonary gradient (DPG), pulmonary vascular resistance (PVR) and transpulmonary gradient (TPG) after LVAD implantation and their impact on survival after LVAD and heart transplantation.

**Results:**

A total of 73 patients who underwent LVAD (HeartMate III) implantation between 2016 and 2022 were retrospectively studied. According to pre-LVAD catheterization, 49 (67.1%) patients had DPG < 7 mmHg and 24 (32.9%) patients had DPG ≥ 7 mmHg. The patients with a pre-VAD DPG ≥ 7 mmHg had higher frequencies of right ventricular (RV) failure (*p* < 0.001), RVAD insertion (*p* < 0.001), need for renal replacement therapy (*p* = 0.002), total mortality (*p* = 0.036) and on-VAD mortality (*p* = 0.04) with a longer ICU stay (*p* = 0.001) compared to the patients with DPG < 7 mmHg. During the follow-up period of 38 (12–60) months, 24 (32.9%) patients died. Pre-LVAD DPG ≥ 7 mmHg (adjusted HR 1.83, 95% CI 1.21–6.341, *p* = 0.039) and post-LVAD DPG ≥ 7 mmHg (adjusted HR 3.824, 95% CI 1.482–14.648, *p* = 0.002) were associated with increased risks of mortality. Neither pre-LVAD TPG ≥ 12 (*p* = 0.505) nor post-LVAD TPG ≥ 12 mmHg (*p* = 0.122) was associated with an increased risk of death. Pre-LVAD PVR ≥ 3 WU had a statistically insignificant risk of mortality (HR 2.35, 95% CI 0.803–6.848, *p* = 0.119) while post-LVAD PVR ≥ 3 WU had an increased risk of death (adjusted HR 2.37, 95% CI 1.241–7.254, *p* = 0.038). For post-transplantation mortality, post-LVAD DPG ≥ 7 mmHg (*p* = 0.55), post-LVAD TPG ≥ 12 mmHg (*p* = 0.85) and PVR ≥ 3 WU (*p* = 0.54) did not have statistically increased risks. The logistic multivariable regression showed that post-LVAD PVR ≥ 3 WU (*p* = 0.013), post-LVAD DPG ≥ 7 mmHg (*p* = 0.026) and RVF (*p* = 0.018) were the predictors of mortality after LVAD implantation. Pre-LVAD DPG ≥ 7 mmHg (*p* < 0.001) and pre-LVAD PVR ≥ 3 WU (*p* = 0.036) were the predictors of RVF after LVAD implantation.

**Conclusions:**

Persistently high DPG was associated with right ventricular failure and mortality after LVAD implantation rather than after heart transplantation. DPG is a better predictor of pulmonary vascular remodeling compared to TPG and PVR. Further larger prospective studies are required in this field due to the growing numbers of patients with advanced heart failure, as possible candidates for LVAD implantation, and limitations of heart transplantation.

**Supplementary Information:**

The online version contains supplementary material available at 10.1186/s43044-023-00428-4.

## Background

The development of pulmonary hypertension (PH) in patients with left-sided heart disease (PH-LHD) is associated with a worse impact on survival [1–3]. It is a complex process that results from persistently high hydrostatic pressures on pulmonary vascular remodeling. The gradually increased pulmonary capillary wedge pressure (PCWP) initially cause a passive elevation of pulmonary venous and arterial pressures. Long-standing PH can cause pulmonary vascular remodeling with endothelial thickening, and fibrosis of the pulmonary vasculature, resulting in post-capillary PH and elevated pulmonary vascular resistance (PVR) [4].

The diastolic pulmonary gradient (DPG), which is the difference between diastolic pulmonary artery pressure (dPAP) and mean PCWP, has been used to differentiate isolated post-capillary PH (IpcPH) with DPG < 7 mmHg from combined post-capillary and pre-capillary PH (CpcPH) with DPG ≥ 7 mmHg [5]. The DPG has been proposed as a better indicator of pulmonary vascular remodeling compared to the PVR (which is mainly affected by compliance of pulmonary vessels) and the transpulmonary gradient (TPG) which is more affected by left atrial pressure and cardiac output [6–8].

Recently, the cutoff levels of the pulmonary hemodynamics and the PVR have been changed according to the 6th World Symposium on Pulmonary Hypertension (WSPH) that released 2022 European Society of Cardiology/European Respiratory Society Guidelines. IpcPH is defined as mPAP > 20 mm Hg, PAWP > 15 mm Hg and PVR ≤ 2WU while CpcPH is defined as mPAP > 20 mm Hg, PAWP > 15 mm Hg and PVR > 2 WU [9].

The left ventricular assist devices (LVADs) are increasingly used for advanced heart failure as a bridge to heart transplantation or as a destination therapy. It has been speculated that LVADs may induce reverse remodeling of the pulmonary vasculature [10–12]. However, there are still few data about the pulmonary hemodynamic changes with LVAD implantation and their impact on candidacy to heart transplantation. The objective of this study was to evaluate the pulmonary hemodynamics changes with LVAD implantation and their impact on survival after LVAD and heart transplantation.

## Methods

### Study design and data collection

This was an observational retrospective cohort study that was approved by the Institutional Review Board of King Faisal Specialist Hospital and was given the reference number (2181248). We enrolled only adult patients ≥ 18 years old who underwent LVAD implantation, between April 2016 and May 2022, with waiving of informed consent due to the retrospective analysis and absence of identifiable data or photographs. All patients recruited had right heart catheterization (RHC) and echocardiography before and after LVAD implantation in our tertiary cardiac center. According to our center policy, the patient selection for LVAD and eligibility for heart transplantation is discussed in the multidisciplinary discussion. For the heart failure patients with reduced ejection fraction (HFrEF) who are dependent on inotropic support or temporary mechanical circulatory support (MCS), durable LVAD is considered as a bridge to transplantation or a destination therapy due to its survival benefit and improvement of quality of life [13, 14]. Heart transplantation is offered to patients with advanced HFrEF despite maximal medical therapy without contraindications due to its survival benefit and improved quality of life [14, 15]. The absolute contraindications included age > 70 years, severe neurological or psychiatric disorders, advanced liver or renal diseases and malignancy. The relative contraindications included significant pulmonary hypertension, reduced pulmonary functions and inability to make a commitment to the transplant team [15]. Data collection was done through the hospital’s electronic records, and there was no loss of follow-up. The demographic, clinical, laboratory, echocardiographic and RHC variables were collected. The primary outcome was all and on-VAD mortality. The secondary outcomes included ICU stay, need for dialysis, right ventricular failure (RVF) and need for right ventricular assist device (RVAD).

### Echocardiography and RHC variables

The recruited patients underwent detailed echocardiographic assessments before and after LVAD implantation. In case of multiple assessments, the last echocardiography pre-LVAD and the first detailed one after LVAD were used for analysis. The variables studied included: the left ventricular end diastolic volume (LV-EDV), the left ventricular end systolic volume (LV-ESV), the left ventricular ejection fraction (LV-EF), the left atrium (LA) diameter, the pulmonary artery systolic pressure (PASP) and the presence of valvular lesions.

For invasive pulmonary hemodynamics assessment, RHC was done before and after LVAD implantation. The cardiac output (CO) was measured by the Fick method. The hemodynamic parameters included systolic pulmonary artery pressure (sPAP), diastolic PAP (dPAP), mean PAP (mPAP), PCWP, PVR and systemic vascular resistance (SVR). The DPG was the difference between dPAP and PCWP while the TPG was the difference between mPAP and PCWP. To calculate the PVR in Wood units (WU), the TPG was divided by the CO [5]. According to pre-LVAD catheterization, the patients studied were divided into the DPG < 7 mmHg and the DPG ≥ 7 mmHg groups.

### Statistical analysis

Data were checked for normality using Shapiro–Wilk and Kolmogorov–Smirnov tests, skewness, kurtosis and plots, and were proved to be deviated from normal distribution so we used the median with interquartile range (IQR) for quantitative data (Additional file 1). Data were summarized using frequency (with percentage) for categorical data using the Statistical Package for the Social Sciences (SPSS) version 28 (IBM Corp., Armonk, NY, USA). Chi-square (*x*^2^) test was used for comparing categorical data. Mann–Whitney test was used for comparing quantitative variables. For comparing serial measurements within each patient, Wilcoxon signed-rank test was used. The Kaplan–Meier method was used to get the survival curves, and the log-rank test was used for comparisons. The Cox proportional hazard analysis was used in the regression models to get the hazards ratios with 95% confidence intervals. Two-sided *p*-values <0.05 were considered statistically significant.

## Results

### Demographic and clinical characteristics

We enrolled 73 patients who underwent LVAD (HeartMate III) implantation with a median age of 43.2 (30.9–54) years and a body mass index of 25.6 (22.1–29.3) kg/m^2^, and 60 (82.2%) of them were males. LVAD implantation was done in 56 (76.7%) patients as a possible bridge to heart transplantation, in 15 (20.5%) patients already listed for transplantation and in 2 (2.7%) patients as a destination therapy. According to pre-LVAD catheterization, 49 (67.1%) patients had a DPG < 7 mmHg, and 24 (32.9%) patients had a DPG ≥ 7 mmHg. The 2 groups had statistically insignificant clinical and laboratory variables before LVAD implantation (Table 1).

**Table 1:** Pre-LVAD characteristics of the patients studied

Variables	All patients (*n* = 73)	Pre-VAD DPG < 7 (*n* = 49, 67.1%)	Pre-VAD DPG ≥ 7 (*n* = 24, 32.9%)	*P* value
Age (years)	43.2 (30.9–54)	43.2 (31.4–54.3)	43.6 (24.4–51.65)	0.74
Gender, male (*n*, %)	60 (82.2)	38 (77.6)	22 (91.7)	0.198
BSA (m^2^)	1.83 (1.68–1.95)	1.8 (1.71–1.95)	1.85 (1.64–2.23)	0.73
BMI (kg/m^2^)	25.6 (22.1–29.3)	25.4 (22.1–28.8)	25.85 (22.35–29.4)	0.76
Smoking (*n*, %)	14 (19.2)	10 (20.4)	4 (16.7)	1
Diabetes mellitus (*n*, %)	31 (42.5)	21 (42.9)	10 (41.7)	0.92
CKD (*n*, %)	13 (17.8)	9 (18.4)	4 (16.7)	1
Pulmonary disease (*n*, %)	5 (6.8)	2 (4.1)	3 (12.5)	0.32
Preoperative AF (*n*, %)	31 (42.5)	19 (38.8)	12 (50)	0.36
Heart disease (*n*, %)				
Idiopathic cardiomyopathy	42 (57.5)	26 (53.1)	16 (66.7)	0.78
Ischemic cardiomyopathy	23 (31.5)	17 (34.7)	6 (25)	
ACHD	3 (4.1)	2 (4.1)	1 (4.2)	
Others	5 (6.8)	4 (8.2)	1 (4.2)	
Time of LVAD after cardiac diagnosis				
< 1 year	15 (20.5)	10 (20.4)	5 (20.8)	0.49
1–2 years	13 (17.8)	7 (14.3)	6 (25)	
> 2 years	45 (61.6)	32 (65.3)	13 (54.2)	
INTERMACS class (*n*, %)				
I	6 (8.2)	4 (8.2)	2 (8.3)	0.73
II	29 (39.7)	20 (40.8)	9 (37.5)	
III	25 (34.2)	18 (36.7)	7 (29.2)	
IV	13 (17.8)	7 (14.3)	6 (25)	
CRT (*n*, %)	14 (19.2)	5 (10.2)	9 (37.5)	0.01
ICD (*n*, %)	49 (67.1)	33 (67.3)	16 (66.7)	0.95
PVD (*n*, %)	1 (1.4)	1 (2)	0	1
Prior stroke (*n*, %)	6 (8.2)	5 (10.2)	1 (4.2)	0.65
Preoperative drugs (*n*, %)				
Beta-blockers	58 (79.5)	40 (81.6)	18 (75)	0.54
Amiodarone	23 (31.5)	13 (26.5)	10 (41.7)	0.19
Spironolactone	51 (69.9)	31 (63.3)	20 (83.3)	0.07
ACEI/ARBs	38 (52.1)	25 (51)	13 (54.2)	0.8
Systemic steroids	3 (4.1)	3 (6.1)	0	0.54
Immune-suppressants	0	0	0	
Warfarin	25 (34.2)	16 (32.7)	9 (37.5)	0.68
Preoperative blood levels				
Creatinine (umol/L)	101 (74–137)	94 (69–138)	102 (77–128.5)	0.52
Bilirubin (umol/L)	25 (11–39)	23 (11–34)	26.5 (14.35–46.4)	0.36
BUN (mg/dL)	10 (6–16)	8.9 (5.7–18)	11.65 (7.9–15.7)	0.27
Albumin (g/L)	38 (35–41.2)	39 (35–41.7)	37.5 (35–40)	0.59
ALT (U/L)	23 (15–45.7)	22 (14.1–44.5)	27.45 (15.3–68)	0.64
AST (U/L)	27 (20–65)	26 (20–65)	29 (19.8–62.5)	0.81
Sodium (mmol/L)	136 (132–138)	136 (133–139)	133.5 (130.5–137)	0.06
Platelets (10^9^/L)	232 (171–296)	239 (173–296)	211 (166–299)	0.78
Hemoglobin (g/L)	113 (101–132)	116 (105–133)	108 (92.5–126.5)	0.12
WBCs (10^9^/L)	6.7 (5.8, 10)	6.7 (5.8–11)	6.9 (5.95–9.45)	0.8

Data were presented as median with the 25th and 75th interquartiles or count with frequency

*BSA* body surface area, *CKD* chronic kidney disease, *BMI* body mass index, *ICD* implantable cardioverter defibrillator, *CRT* cardiac resynchronization therapy, *ACHD* adult congenital heart disease, *AF* atrial fibrillation, *ACEI* angiotensin-converting enzyme inhibitors, *ARBs* angiotensin receptor blockers, *PVD* peripheral vascular disease, *BUN* blood urea nitrogen, *AST* aspartate transferase, *ALT* alanine transaminase

### Echocardiographic and RHC data

Before LVAD implantation, the patients with the DPG < 7 mmHg had lesser LV-EDV (*p* = 0.04), LV-ESV (*p* = 0.047) and PASP (*p* < 0.001) compared to the patients with DPG ≥ 7 without statistically significant differences regarding other echocardiographic variables. After LVAD implantation, the patients with the DPG < 7 mmHg had lesser EDV (*p* = 0.002), ESV (*p* = 0.005), PASP (*p* < 0.041) and LA diameter (*p* = 0.04) compared to those with DPG ≥ 7. The DPG ≥ 7 group had higher frequencies of moderate/severe mitral valve regurgitation (*p* = 0.005) and severe tricuspid regurgitation (*p* = 0.014) (Table 2).

**Table 2:** Echocardiographic variables of the patients studied

Variables	All patients (*n* = 73)	Pre-VAD DPG < 7 (*n* = 49, 67.1%)	Pre-VAD DPG ≥ 7 (*n* = 24, 32.9%)	*P* value
Pre-LVAD echocardiography				
LV-EDV (ml)	241.4 (189.2–299.8)	218.9 (180–273)	265.45 (196.7–384.05)	0.04
LV-ESV (ml)	191.8 (140–247.4)	172.9 (136.3–236.8)	211.9 (161.65–315.75)	0.047
LV-EF (%)	20.2 (14–25.7)	20.5 (14.4–26.8)	19.05 (12.95–25.2)	0.46
LA diameter (cm)	5.1 (4.5–5.4)	4.7 (4.4–5.4)	5.3 (4.9–5.95)	0.27
Cardiac output (L/min)	3.7 (2.89–4.3)	3.7 (2.85–4.3)	3.9 (2.89–4.5)	0.622
Cardiac index (L/min per m^2^)	1.89 (1.52–2.3)	1.88 (1.56–2.35)	1.9 (1.45–2.3)	0.9
Estimated PASP (mmHg)	55 (45–60)	50 (44–63)	68 (58–76)	< 0.001
Mitral regurgitation (*n*, %)				
No MR	0	0	0	0.08
Mild MR	14 (19.2)	11 (22.4)	3 (12.5)	
Moderate MR	33 (45.2)	25 (51)	8 (33.3)	
Severe MR	26 (35.6)	13 (26.5)	13 (54.2)	
Tricuspid regurgitation (*n*, %)				
No TR	0	0	0	0.24
Mild TR	14 (19.2)	12 (24.5)	2 (8.3)	
Moderate TR	32 (43.8)	21 (42.9)	11 (45.8)	
Severe TR	27 (37)	16 (32.7)	11 (45.8)	
Aortic regurgitation (*n*, %)				
No AR	57 (78.1)	40 (81.6)	17 (70.8)	0.29
Mild AR	16 (21.9)	9 (18.4)	7 (29.2)	
Post-LVAD echocardiography				
LV-EDV (ml)	174.75 (120.5–211.5)	161.35 (106.05–193.9)	210.45 (151.45–243.7)	0.002
LV-ESV (ml)	128.6 (86.4–167.7)	110.9 (71.4–154.5)	155.3 (120.75–188.05)	0.005
LV-EF (%)	23.4 (18.1–30.8)	25 (20–35.8)	20.7 (15–23.9)	0.03
LA diameter (cm)	4.5 (4.3–5)	4.5 (4.2–4.8)	4.8 (4.4–5.2)	0.04
Estimated PASP (mmHg)	35 (30–42.5)	35 (30–40)	40 (35–50)	0.041
Mitral regurgitation (*n*, %)				
No MR	8 (10.9)	6 (12.2)	2 (8.3)	0.005
Mild MR	53 (72.6)	40 (81.6)	13 (54.2)	
Moderate MR	11 (15.1)	3 (6.1)	8 (33.3)	
Severe MR	1 (1.4)	0	1 (4.2)	
Tricuspid regurgitation (*n*, %)				
No TR	1 (1.4)	0	1 (4.2)	0.014
Mild TR	48 (65.8)	36 (73.5)	12 (50)	
Moderate TR	16 (21.9)	11 (22.4)	5 (20.8)	
Severe TR	8 (11)	2 (4.1)	6 (25)	
Aortic regurgitation (*n*, %)				
No AR	49 (67.1)	35 (71.4)	14 (58.3)	0.45
Mild AR	23 (31.5)	13 (26.5)	10 (41.7)	
Moderate AR	1 (1.4)	1 (2)	0	

Data were presented as median with the 25th and 75th interquartiles or count with frequency

*LV-EDV* left ventricular end diastolic volume, *LV-ESV* left ventricular end systolic volume, *LV-EF* left ventricular ejection fraction, *LA* left atrium, *PASP* pulmonary artery systolic pressure, *MR* mitral regurgitation, *AR* aortic regurgitation, *TR* tricuspid regurgitation

Before LVAD implantation, the patients with DPG < 7 mmHg had lesser sPAP (*p* < 0.001), dPAP (*p* < 0.001), mPAP (*p* < 0.001), TPG (*p* < 0.001) and PVR (*p* < 0.001) compared to the patients with DPG ≥ 7 groups. After LVAD implantation, the patients with DPG < 7 mmHg had lesser sPAP (*p* < 0.001), dPAP (*p* = 0.012), mPAP (*p* = 0.002), TPG (*p* = 0.01) and PVR (*p* = 0.019) compared to those with DPG ≥ 7 mmHg (Table 3).

**Table 3:** Pulmonary hemodynamics of the patients studied

Variables	All patients	Pre-VAD DPG < 7	Pre-VAD DPG ≥ 7	*P* value
*Pre-LVAD RHC*				
sPAP (mmHg)	58 (47–68)	54 (44–63)	68 (58.5–76)	< 0.001
dPAP (mmHg)	31 (25–36)	28 (23–33)	35 (31.5–43)	< 0.001
mPAP (mmHg)	42 (33–46)	38 (31–43)	46.5 (42–52.5)	< 0.001
PCWP (mmHg)	26 (21–31)	25 (20–31)	27 (22.5–35)	0.182
Fick cardiac output (L/min)	3.27 (2.81–3.9)	3.26 (2.81–3.68)	3.83 (2.75–4.23)	0.33
Fick cardiac index (L/min per m^2^)	1.83 (1.63–2.13)	1.81 (1.63–2.08)	2.03 (1.44–2.16)	0.59
DPG (mmHg)	4 (2–7)	2 (1–4)	8 (7–10)	< 0.001
TPG (mmHg)	13 (10–17)	11 (9–14)	18 (14.5–24)	< 0.001
PVR (dynes-sec/cm^−5^)	258.15 (208.1–405.3)	235.3 (185.5–309.3)	397.5 (252.4–571.4)	< 0.001
PVR index (dynes-sec/cm^−5^/m^2^)	511.35 (376.7–763.8)	450.8 (313.5–578.1)	819.5 (513.2–1082.8)	< 0.001
PVR (WU)	3.66 (2.94–4.78)	3.3 (2.6–3.87)	4.84 (3.5–6.39)	< 0.001
SVR (dynes-sec/cm^−5^)	1521.5 (1283.4–2018.5)	1517.3 (1264.7–1936.1)	1528.5 (1293.4–2112)	0.51
SVR index (dynes-sec/cm^−5^/m^2^)	2832.8 (2463.9–3427.5)	2823.7 (2296.2–3262.6)	3079.3 (2519.3–3967.7)	0.124
TPR (dynes-sec/cm^−5^)	916.01 (721–1071.9)	869.7 (700.2–1027.1)	965.2 (832.5–1753)	0.074
TPR index (dynes-sec/cm^−5^/m^2^)	1705.6 (1294.7–2017.4)	1586.1 (1271.7–1920)	1913.3 (1677.9–2666.6)	0.016
PVR/SVR ratio	0.18 (0.14–0.25)	0.16 (0.13–0.23)	0.23 (0.19–0.32)	0.002
*Post-LVAD RHC*				
sPAP (mmHg)	41 (34–48)	37 (32.5–43)	47 (42–62)	< 0.001
dPAP (mmHg)	21 (18–26)	19.5 (15.5–23.5)	24 (20–32)	0.012
mPAP (mmHg)	28 (24–34)	26.5 (23–31)	32 (27–42)	0.002
PCWP (mmHg)	18 (15–21)	17 (14–20)	19 (16–27)	0.038
Fick cardiac output (L/min)	4.24 (3.8–4.87)	4.22 (3.78–4.86)	4.24 (3.94–5.2)	0.45
Fick cardiac index (L/min per m^2^)	2.29 (1.94–2.54)	2.3 (1.97–2.5)	2.28 (1.87–2.7)	0.95
DPG (mmHg)	3 (2–5)	3 (2–5)	4 (2–7)	0.09
TPG (mmHg)	10 (8–13)	10 (8–12.5)	12 (9–16)	0.01
PVR (dynes-sec/cm^−5^)	168 (129.4–234.7)	158.85 (126.5–216.65)	205.1 (148.2–292.7)	0.023
PVR index (dynes-sec/cm^−5^/m^2^)	334.1 (257.1–437.1)	320.1 (223.98–422.8)	372.3 (316.4–539.5)	0.054
PVR (WU)	2.44 (1.87–3.13)	2.26 (1.81–2.91)	2.84 (2.36–3.41)	0.019
SVR (dynes-sec/cm^−5^)	1455.4 (1240.2–1736.3)	1440 (1215.8–1790.7)	1490.6 (1323.5–1626.02)	0.85
SVR index (dynes-sec/cm^−5^/m^2^)	2621.3 (2173.2–3141.8)	2617.8 (2158.4–3141.8)	2714.2 (2531.6–3126)	0.67
TPR (dynes-sec/cm^−5^)	562.8 (412.9–743.7)	529.6 (410.7–743.7)	631.8 (509.2–738.5)	0.38
TPR index (dynes-sec/cm^−5^/m^2^)	962.4 (790.3–1360)	919.9 (763.7–1283.6)	1262.4 (943.1–1575.8)	0.033
PVR/SVR ratio	0.13 (0.11–0.18)	0.12 (0.11–0.15)	0.2 (0.13–0.24)	0.026

Data were presented as median with the 25th and 75th interquartiles

*DPG* diastolic pulmonary gradient, *TPG* transpulmonary gradient, *sPAP* systolic pulmonary artery pressure, *dPAP* diastolic pulmonary artery pressure, *mPAP* mean pulmonary artery pressure, *PCWP* pulmonary capillary wedge pressure, *PVR* pulmonary vascular resistance, *SVR* systemic vascular resistance, *TPR* transpulmonary resistance, *WU* Wood unit

**Fig. 1:**
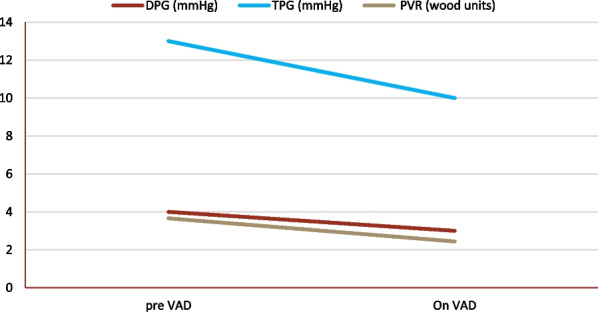
Changes of DPG (*p* = 0.097), TPG (*p* < 0.001) and PVR (*p* < 0.001) after LVAD implantation

Post-LVAD assessments showed a significant decrease in EDV (*p* < 0.001), ESV (*p* < 0.001) and LA diameter (*p* < 0.001) compared to the pre-LVAD measurements. The LVAD was associated with the decrease in the sPAP (*p* < 0.001), dPAP (*p* < 0.001), mPAP (*p* < 0.001), PCWP (*p* < 0.001), DPG (*p* = 0.097), TPG (p < 0.001) and PVR (p < 0.001) (Table 4; Fig. 1).

**Table 4:** Echocardiographic and pulmonary hemodynamics changes after LVAD

Variables	Pre-LVAD	Post-LVAD	*P* value
LV-EDV (ml)	241.4 (189.2–299.8)	174.75 (120.5–211.5)	< 0.001
LV-ESV (ml)	191.8 (140–247.4)	128.6 (86.4–167.7)	< 0.001
LA diameter (cm)	5.1 (4.5–5.4)	4.5 (4.3–5)	< 0.001
sPAP (mmHg)	58 (47–68)	41 (34–48)	< 0.001
dPAP (mmHg)	31 (25–36)	21 (18–26)	< 0.001
mPAP (mmHg)	42 (33–46)	28 (24–34)	< 0.001
PCWP (mmHg)	26 (21–31)	18 (15–21)	< 0.001
DPG (mmHg)	4 (2–7)	3 (2–5)	0.097
TPG (mmHg)	13 (10–17)	10 (8–13)	< 0.001
PVR (WU)	3.66 (2.94–4.78)	2.44 (1.87–3.13)	< 0.001

Data were presented as median with the 25th and 75th interquartiles

*LV-EDV* left ventricular end diastolic volume, LV-ESV: left ventricular end systolic volume, *LA* left atrium, *DPG* diastolic pulmonary gradient, *TPG* transpulmonary gradient, *sPAP* systolic pulmonary artery pressure, *dPAP* diastolic pulmonary artery pressure, *mPAP* mean pulmonary artery pressure, *PCWP* pulmonary capillary wedge pressure, *PVR* pulmonary vascular resistance

### Outcomes and survival analysis

The patients with a pre-VAD DPG ≥ 7 mmHg had higher frequencies of RVF (*p* < 0.001), RVAD insertion (*p* < 0.001), acute kidney injury (*p* < 0.001), new need for renal replacement therapy (*p* = 0.002), total mortality (*p* = 0.036) and on-VAD mortality (*p* = 0.04) with a longer ICU stay (*p* = 0.001) compared to the patients with DPG < 7 mmHg (Table 5).

**Table 5:** Outcomes of the cohort analysis

Variables	All patients (*n* = 73)	Pre-VAD DPG < 7 (*n* = 49, 67.1%)	Pre-VAD DPG ≥ 7 (*n* = 24, 32.9%)	*P* value
RVF (*n*, %)	24 (32.9)	3 (6.1)	21 (87.5)	< 0.001
RVAD insertion (*n*, %)	13 (17.8)	2 (4.1)	11 (45.8)	< 0.001
AKI (*n*, %)	43 (58.9)	21 (42.9)	22 (91.7)	< 0.001
Need for CRRT (*n*, %)	20 (27.4)	8 (16.3)	12 (50)	0.002
Exploration for bleeding (*n*, %)	13 (17.8)	6 (12.2)	7 (29.2)	0.11
Cerebrovascular stroke (*n*, %)	6 (8.2)	3 (6.1)	3 (12.5)	0.39
Tracheostomy (*n*, %)	12 (16.4)	7 (14.3)	5 (20.8)	0.51
ICU days	9 (8–16)	9 (8–16)	24 (15–42.5)	0.001
Milrinone days	4 (3–6)	4 (3–6)	13 (6–16)	< 0.001
Inhaled NO days	3 (2–4)	3 (2–4)	9.5 (4.5–14.5)	< 0.001
Ventilator days	2 (1–3)	2 (1–3)	6.5 (3–26)	< 0.001
Septicemia (*n*, %)	9 (12.3)	4 (8.2)	5 (20.8)	0.14
Total mortality (*n*, %)	24 (32.9)	13 (26.5)	11 (45.8)	0.036
On-VAD mortality (*n*, %)	18 (24.7)	10 (20.4)	8 (33.3)	0.04
Transplantation (*n*, %)	17 (23.3)	10 (20.4)	7 (29.2)	0.41
Mortality after transplantation (*n*, %)	6 (35.3)	3 (30)	3 (42.9)	0.64

Data were presented as count with frequency or median with the 25th and 75th interquartiles

*RVF* right ventricular failure, *RVAD* right ventricular assist device, *AKI* acute kidney injury, *CRRT* continuous renal replacement therapy, *ICU* intensive care unit, *NO* nitric oxide

During the follow-up period of 38 (12–60) months, 17 (23.3%) patients had heart transplantation after a median of 10 (6–15) months and 24 (32.9%) patients died. Cox proportional hazard regression revealed that pre-LVAD DPG ≥ 7 mmHg (*p* = 0.036) and post-LVAD DPG ≥ 7 mmHg (*p* = 0.005) were associated with increased risks of mortality.

Pre-LVAD TPG ≥ 12 mmHg (*p* = 0.505) and post-LVAD TPG ≥ 12 mmHg (*p* = 0.122) did not have significantly increased risks of death. Pre-LVAD PVR ≥ 3 WU had a statistically insignificant risk of mortality (*p* = 0.119) while post-LVAD PVR ≥ 3 WU had an increased risk of death (HR 2.56, 95% CI 1.117–5.848, *p* = 0.026) (Table 6).

**Table 6:** Risks of mortality according to pulmonary hemodynamics

Variables	HR	95% CI	*P* value
Pre-LVAD DPG ≥ 7 mmHg			
Crude	2.048	1.28–4.518	0.036
Adjusted	1.83	1.21–6.341	0.039
Pre-LVAD TPG ≥ 12 mmHg	1.321	0.583–2.992	0.505
Pre-LVAD PVR ≥ 3 WU	2.35	0.803–6.848	0.119
Pre-LVAD PVR ≥ 2 WU	1.61	0.62–5.17	0.42
Post-LVAD DPG ≥ 7 mmHg			
Crude	4.243	1.561–11.538	0.005
Adjusted	3.824	1.482–14.648	0.002
Post-LVAD TPG ≥ 12 mmHg	1.91	0.841–4.338	0.122
Post-LVAD PVR ≥ 3 WU			
Crude	2.56	1.117–5.848	0.026
Adjusted	2.37	1.241–7.254	0.038
Pre-LVAD PVR ≥ 2 WU	1.74	0.97–6.12	0.057
Pre-transplantation DPG ≥ 7 mmHg	1.94	0.22–16.75	0.548
Pre-transplantation TPG ≥ 12 mmHg	1.174	0.215–6.42	0.85
Pre-transplantation PVR ≥ 3 WU	1.937	0.22–16.747	0.55
Pre-transplantation PVR ≥ 2 WU	1.31	0.21–8.37	0.58
Post-transplantation DPG ≥ 7 mmHg (T3)	4.16	0.37–46.68	0.25
Post-transplantation TPG ≥ 12 mmHg (T3)	6.35	0.358–112.8	0.21
Post-transplantation PVR ≥ 3 WU (T3)	8.12	0.49–134.1	0.14
Post-transplantation DPG ≥ 7 mmHg (T6)	0.033	0.001–495.2	0.64
Post-transplantation TPG ≥ 12 mmHg (T6)	0.039	0.001–219.85	0.722
Post-transplantation PVR ≥ 3 WU (T6)	0.044	0.000–1639.42	0.844
Post-transplantation DPG ≥ 7 mmHg (T12)	0.04	0.000–6575.52	0.77
Post-transplantation TPG ≥ 12 mmHg (T12)	0.04	0.000–1659.4	0.84
Post-transplantation PVR ≥ 3 WU (T12)	0.04	0.000–1659.4	0.84

Pre-transplantation variables are the post-LVAD variables in patients who underwent heart transplantation. Adjustment of HR was done for age, gender, atrial fibrillation, CRT, BMI and mitral regurgitation

For post-transplantation mortality, post-LVAD DPG ≥ 7 mmHg (*p* = 0.55), post-LVAD TPG ≥ 12 mmHg (*p* = 0.85) and PVR ≥ 3 WU (*p* = 0.54) did not have statistically increased risks of death in our cohort (Table 6).

The logistic multivariable regression showed that post-LVAD PVR ≥ 3 WU (*p* = 0.013), post-LVAD DPG ≥ 7 mmHg (*p* = 0.026) and RVF (*p* = 0.018) were the predictors of mortality after LVAD. Pre-LVAD DPG ≥ 7 mmHg (*p* < 0.001) and pre-LVAD PVR ≥ 3 WU (*p* = 0.036) were the predictors of RVF after LVAD implantation (Table 7).

**Table 7:** Logistic multivariable regression for mortality and RVF

Variables	OR	95% CI	*P* value
Post-LVAD mortality			
Post-LVAD PVR ≥ 3 WU	4.995	1.405–17.756	0.013
Post-LVAD DPG ≥ 7 mmHg	2.37	1.31–24.8	0.026
RVF failure	5.59	1.564–19.98	0.018
Post-LVAD MR	1.04	0.82–18.26	0.72
Post-LVAD RVF			
Pre-LVAD DPG ≥ 7 mmHg	4.31	3.29–19.572	< 0.001
Pre-LVAD PVR ≥ 3 WU	2.49	1.92–17.32	0.036
Pre-LVAD TPG ≥ 12 mmHg	1.27	0.682–24.28	0.42
Pre-LVAD severe MR	1.32	0.417–27.38	0.47

*PVR* pulmonary vascular resistance, *DPG* diastolic pulmonary gradient, *TPG* transpulmonary gradient, *OR* odds ratio, *CI* confidence interval, *WU* wood unit, *RVF* right ventricular failure, *MR* mitral regurgitation

**Fig. 2:**
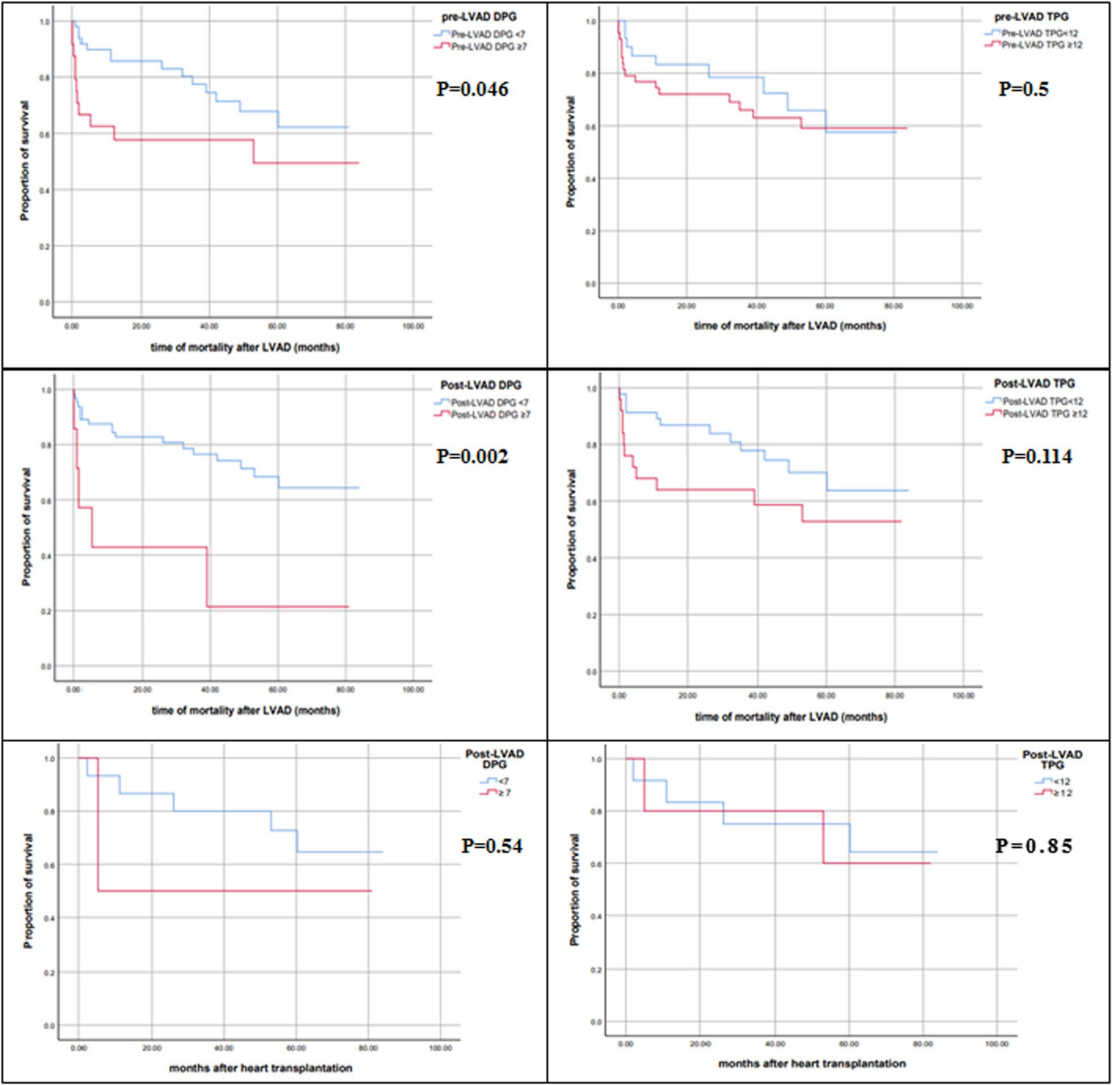
Kaplan–Meier survival curves of the patients studied according to DPG and TPG with the log-rank *p*-values

**Fig. 3:**
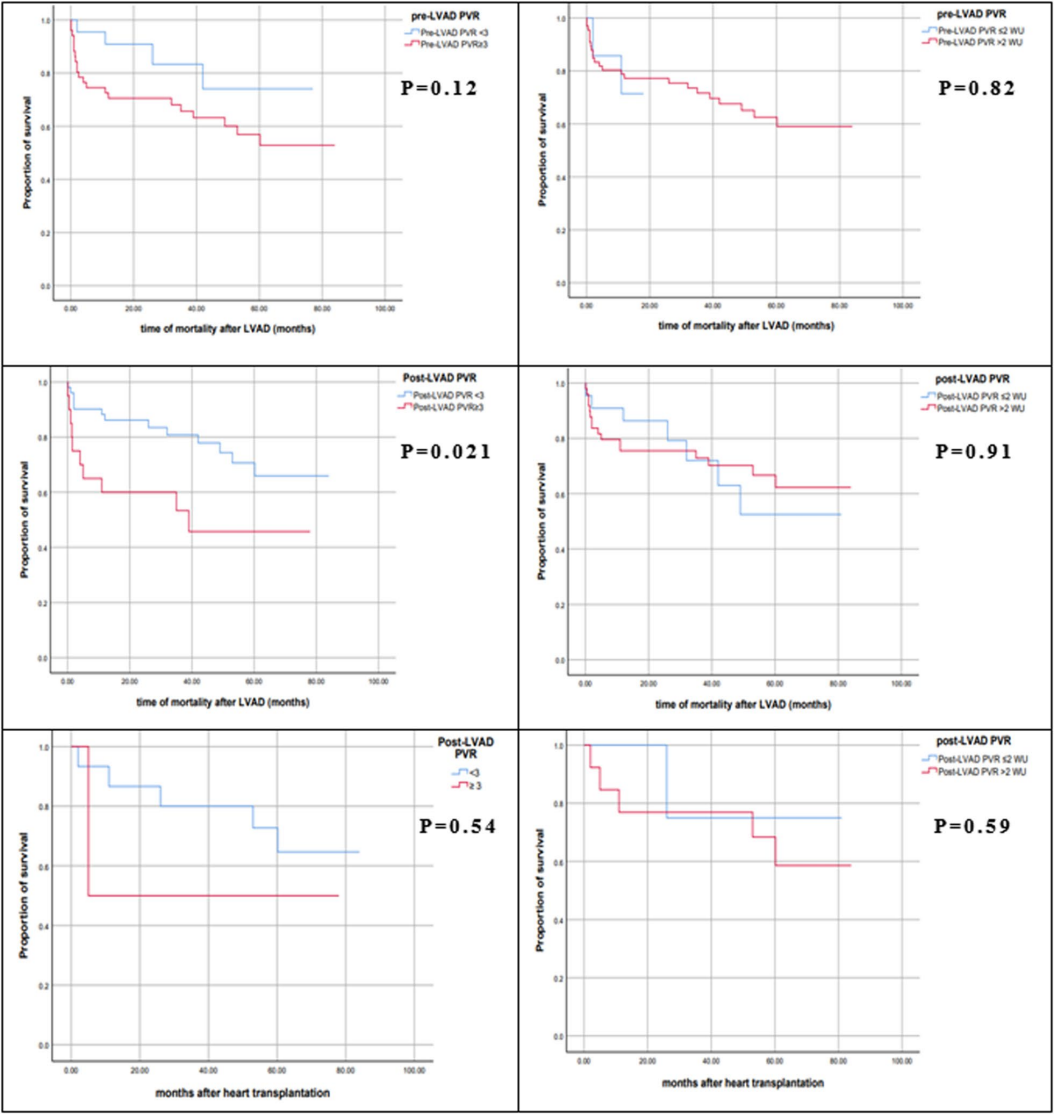
Kaplan–Meier survival curves of the patients studied according to PVR with the log-rank *p*-values

Survival analyses were graphed by Kaplan–Meier curves with log-rank *p* values according to pre- and post-LVAD DPG, TPG and PVR (Figs. 2, 3).

## Discussion

Our main findings were that the high pre-LVAD DPG was associated with increased occurrence of RV failure and mortality. Persistently high DPG after LVAD carried an increased risk of decreased survival on LVAD, but was not associated with post-transplantation mortality. LVAD insertion was associated with statistically significant improvement of the pulmonary hemodynamic parameters except DPG. Neither pre- nor post-VAD TPG ≥ 12 mmHg was associated with mortality after LVAD or transplantation. Post-LVAD elevated PVR was associated with an increased risk of post-LVAD rather than post-transplantation mortality.

The LVAD implantation for patients with advanced HFrEF produces left ventricular unloading and improves the pulmonary hemodynamic variables resulting in improvement of survival, quality of life, organs perfusion and functional capacity of the patients [16, 17]. In our cohort analysis, the LVAD implantation was associated with decreased left ventricular volumes, decreased severity of mitral regurgitation and improvement of pulmonary hemodynamic variables. Recently, Grupper et al. [18] have studied 85 adult patients with LVAD (HeartMate II and III) and reported the improvement of pulmonary hemodynamics and echocardiographic parameters after LVAD regardless of the pre-LVAD PVR. Despite the LVAD decreases the severity of MR and pulmonary pressures, RVF may occur due to increased preload, loss of the ventricular interdependence and left-sided deviation of septum with possible right ventricular dilatation and worsening of tricuspid regurgitation [19]. RVF occurred in 32.9% of our study patients and almost half of them required the right ventricular assist device (RVAD). The occurrence of RVF and RVAD insertion were more frequent in the group of elevated DPG. Cox proportional and logistic multivariable regressions showed that elevated DPG and PVR were the independent predictors of RVF and mortality after LVAD.

The DPG was proposed as a better indicator of pulmonary vascular remodeling compared to the PVR (which is mainly affected by compliance of pulmonary vessels) and the TPG which is more affected by left atrial pressure and cardiac output [6–8]. The cutoff of 7 mmHg was used to differentiate IpcPH with DPG < 7 mmHg from CpcPH with DPG ≥ 7 mmHg [5]. There are still conflicting results about the prognostic value of DPG in the patients with heart failure, post-LVAD insertion and recipients of heart transplantation. Gerges et al. [8] found that elevated DPG > 7 mmHg was associated with a significant pulmonary vascular remodeling and a worse prognosis in patients with TPG > 12 mmHg. Tatsuro Ibe et al. [20] studied 164 patients with PH-LHD and found that the DPG was a more sensitive predictor to worse clinical outcomes compared to the TPG. However, Tampakakis et al. [21] conducted a retrospective analysis of 1236 patients and found that the DPG was not associated with mortality at different cutoff points including 7 mmHg.

Our cohort analysis showed that pre-LVAD high DPG was significantly associated with RVF and mortality. Alnsasra et al. [22] retrospectively reviewed 268 patients with pre-LVAD RHC and found that pre-VAD DPG ≥ 7 mmHg was associated with RVF without a survival difference. Alnsasra et al. [22] did not follow the DPG after LVAD insertion to detect the changes in the pulmonary vascular parameters and any potential impact on survival. In our analysis, the decline of DPG after LVAD insertion was not statistically significant like the decline in TPG and PVR and this may explain the ability of DPG to predict mortality after LVAD. Thenappan et al. [23] investigated the effect of LVAD on DPG in 116 patients and found that 42% of them were non-responders with a persistent DPG > 7 mmHg. Imamura et al. [24] prospectively studied 92 patients with LVAD and found DPG > 5 mmHg was associated with RV deterioration and worse outcomes. We could not find an association between DPG and post-transplantation mortality. Ryan et al. [25] conducted a large retrospective analysis of 5827 recipients of heart transplantation with pulmonary hypertension and concluded that the DPG at different cutoff points was not associated with post-transplantation survival.

We found a statistically significant decrease in the PVR after LVAD insertion and post-LVAD PVR ≥ 3 WU was associated with an increased risk of post-LVAD rather than post-transplantation mortality. LVAD insertion significantly decreased the PVR and TPG compared to medical therapy and increased the candidacy to transplantation but without a survival benefit [26]. LVAD insertion was found to decrease the fixed PH with a high PVR and achieved similar survival rates after heart transplantation compared to those without high PVR [27, 28]. Recently, Selim et al. [29] have retrospectively studied 51 patients with high PVR subjected to LVAD and reported the significant decrease in the PVR after LVAD implantation that persisted after heart transplantation for 1-year follow-up. Tsukashita et al. [30] studied 227 recipients of heart transplantation and found that despite normalization of PVR with LVAD, the patients with an initially high PVR had a greater hospital mortality but a similar 3-year mortality.

According to our study, LVAD significantly decreased the TPG but neither pre- nor post-LVAD was associated with mortality after LVAD or transplantation. Mikus et al. [31] reported that LVAD decreased the TPG and PVR and made 63 patients eligible for transplantation. Alnsasra et al. [22] did not find any association between TPG and RV failure or mortality after LVAD insertion. However, Uriel et al. [32] reported that the elevated TPG rather than PVR negatively affects post-transplantation survival in LVAD patients.

Finally, LVAD implantation improves pulmonary hemodynamics by mechanical unloading. Additionally, Thompson et al. [33] reported the significant decrease in plasma levels of endothlin-1, which is a strong vasoconstrictor after LVAD implantation for patients with advanced heart failure. Saidi et al. [34] retrospectively analyzed 38 patients who receive heart transplantation after LVAD support and reported the improvement of pulmonary hemodynamics with both pulsatile and continuous flow LVADs that were sustained for 3–5 years after transplantation.

## Conclusions

Persistently high DPG was associated with right ventricular failure and mortality after LVAD implantation rather than after heart transplantation. DPG is a better predictor of pulmonary vascular remodeling compared to TPG and PVR. Further larger prospective studies are required in this field due to the growing numbers of patients with advanced heart failure, as possible candidates for LVAD implantation, and limitations of heart transplantation.

### Limitations of the study

Our study was a single-center work with retrospective analysis of a relatively medium-sized cohort. The results were underpowered due to the sample size. All patients enrolled had continuous flow pump HeartMate III device which limit generalizability to other brands of LVAD.

### Supplementary Information

**Additional file 1.** Normality testing of the data.

## Data Availability

The data of the study are available with the corresponding author.

## Abbreviations

AKI: Acute kidney injury
CKD: Chronic kidney disease
CRRT: Continuous renal replacement therapy
CpcPH: Combined post-capillary and pre-capillary pulmonary hypertension
IpcPH: Isolated post-capillary PH
INTERMACS: Interagency Registry for Mechanically Assisted Circulatory Support
DPG: Diastolic pulmonary gradient
HFrEF: Heart failure with reduced ejection fraction
LVAD: Left ventricular assist device
LV-EDV: Left ventricular end diastolic volume
LV-ESV: Left ventricular end systolic volume
LV-EF: Left ventricular ejection fraction
LA: Left atrium
PASP: Pulmonary artery systolic pressure
sPAP: Systolic pulmonary artery pressure
dPAP: Diastolic pulmonary artery pressure
mPAP: Mean pulmonary artery pressure
RVF: Right ventricular failure
RVAD: Right ventricular assist device
PCWP: Pulmonary capillary wedge pressure
PVR: Pulmonary vascular resistance
SVR: Systemic vascular resistance
TPR: Transpulmonary resistance
TPG: Transpulmonary gradient
WU: Wood unit
